# A linear interpretable model for drug target prediction

**DOI:** 10.1093/bioinformatics/btag428

**Published:** 2026-08-21

**Authors:** Santiago Noto, Santiago Ferreyra, Ruben Jimenez, Diego Galeano, Alberto Paccanaro

**Affiliations:** School of Applied Mathematics, Fundação Getulio Vargas, Rio de Janeiro, 22250-900, Brazil; Department of Computer Science, Centre for Systems and Synthetic Biology, Royal Holloway, University of London, Egham Hill, Egham, TW20 0EX, United Kingdom; School of Applied Mathematics, Fundação Getulio Vargas, Rio de Janeiro, 22250-900, Brazil; School of Applied Mathematics, Fundação Getulio Vargas, Rio de Janeiro, 22250-900, Brazil; Department of Computer Science, Centre for Systems and Synthetic Biology, Royal Holloway, University of London, Egham Hill, Egham, TW20 0EX, United Kingdom; School of Applied Mathematics, Fundação Getulio Vargas, Rio de Janeiro, 22250-900, Brazil; Department of Computer Science, Centre for Systems and Synthetic Biology, Royal Holloway, University of London, Egham Hill, Egham, TW20 0EX, United Kingdom; School of Applied Mathematics, Fundação Getulio Vargas, Rio de Janeiro, 22250-900, Brazil; Department of Computer Science, Centre for Systems and Synthetic Biology, Royal Holloway, University of London, Egham Hill, Egham, TW20 0EX, United Kingdom

## Abstract

**Motivation:**

Identifying drug targets is fundamental in drug development, both for discovering new therapies and for ensuring effective and safe treatments. Drug-target interactions (DTIs) have been predicted using machine learning approaches that integrate heterogeneous data; however, often these models are complex and lack interpretability.

**Results:**

We investigate whether a simple, fully interpretable linear model can achieve competitive performance for DTI prediction. We propose Linear Interpretable Drug-Target Interaction (LI-DTI), a prediction model inspired by recommender systems. LI-DTI learns from different drug-drug and target-target similarity matrices and provides interpretable predictions as a linear combination of these similarity measures. We show that LI-DTI can recover DTIs even when drugs or targets have no previously known interactions, across multiple cross-validation settings. We further evaluate performance while mitigating potential bias arising from high chemical similarity between drugs or sequence similarity between targets. Finally, we assess LI-DTI in a prospective evaluation, training on DTIs present in DrugBank from 2011 and testing on interactions added through 2022. Across all evaluations, LI-DTI achieves state-of-the-art performance while producing interpretable predictions. For practical use, we provide a web-based tool that enables users to visualize individual LI-DTI predictions for DrugBank (2025) and inspect the biological evidence underlying them. Our results indicate that simple linear models with well-curated similarity features can deliver robust and interpretable DTI predictions, facilitating hypothesis generation and downstream experimental prioritization.

**Availability and implementation:**

Code and data available at https://github.com/paccanarolab/LI-DTI. Web tool available at https://paccanarolab.org/lidtiweb/.

## 1 Introduction

The identification of drug-target interactions (DTI) is a critical step in the drug discovery process. Experimental approaches for identifying targets of small molecules by selective binding are expensive and time-consuming and often fail to identify the full set of relevant DTIs. For this reason, many drug targets remain unknown, and a wide range of computational approaches have been developed to predict them. Among these methods, docking simulation approaches model drug-protein interactions considering the 3D structure of the proteins, but they are computationally expensive, often requiring expert knowledge.

Machine learning approaches typically frame DTI prediction as a binary classification task, where the goal is to predict whether a drug interacts or not with a target. These methods integrate heterogeneous information about individual drugs and targets, often in the form of similarity measures. Early machine learning methods calculated similarities based on molecular features of drugs and targets, e.g. chemical similarity between drugs, sequence similarity between known targets, or information about drug side effects.

More recent approaches integrate information on individual drugs and targets together with known DTIs and are often based on the idea of learning a low-dimensional representation for each drug and target. For instance, Luo *et al.* (2017) introduced DTINet, which constructs a heterogeneous network where nodes represent drugs, targets, diseases and side effects, and edges capture known associations between them. To derive informative representations from this complex graph, DTINet applies diffusion component analysis to learn low-dimensional feature vectors for drugs and targets. These embeddings are then integrated into a matrix completion framework to predict novel DTIs. NeoDTI extends this approach by jointly learning node embeddings and the matrix completion model in an end-to-end deep learning framework, resulting in improved predictive performance (Wan *et al.* 2019). HampDTI introduces an automatic meta-path learning strategy that identifies and weights informative relational patterns, improving the capture of biologically meaningful interactions (Wang *et al.* 2022). Ye *et al.* (2021) proposed KGE_NFM, a heterogeneous knowledge graph approach that captures interaction patterns based on known associations.

Other recent methods, such as MolTrans (Huang *et al.* 2021) and DrugBAN (Bai *et al.* 2023), address a related but not identical problem. These methods are typically trained and evaluated on large-scale binding-affinity datasets derived from BindingDB and ChEMBL, where interactions are defined by thresholding heterogeneous measurements such as Ki, Kd, IC50 and EC50 (Chatterjee *et al.* 2023). In contrast, similarly to DTINet, NeoDTI, HampDTI and KGE_NFM, here we focus on curated DTIs from DrugBank, which provides drug-target annotations supported by expert evaluation and literature evidence. Additional discussion of these differences is provided in Supplementary Materials.

An important limitation of these machine learning models is that they are not interpretable. Interpretability allows us to shed some light on the rationale behind the predictions made by a system, making it possible to assess the contribution of different inputs to a given prediction. This is crucial for the drug target prediction problem, whose objective is to recommend lists of drug targets to be tested experimentally. An interpretable system would allow scientists to analyze model predictions in terms of the underlying biological evidence, thus helping them direct their experiments. However, in these earlier models, interpretability is difficult to achieve due to the complexity of the models, which limits our ability to understand both the model’s decision process and the biological factors driving individual predictions.

In this work, our aim is to test whether a machine learning model that is significantly simpler than the ones that have been proposed in the literature could achieve state-of-the-art predictions. We focus on linear models, due to their ability to provide clear and straightforward interpretability, since their predictions are linear combinations of the inputs. Here, interpretability refers to the ability to disentangle the contribution of different similarity sources to the predictions, rather than to physicochemical explanations of molecular interactions. We were motivated to explore linear models by our previous work (Galeano *et al.* 2020), where we showed that associations between drugs and side effects can be effectively predicted using simple interpretable linear models.

We propose Linear Interpretable Drug-Target Interaction (LI-DTI), a linear approach for DTI prediction. LI-DTI combines two separate linear models, one for drugs and one for targets, together with information from known DTIs. Each linear model integrates chemical, biological, and pharmacological information about drugs and targets expressed in the form of similarity matrices. The model is entirely linear, thus allowing us to easily interpret its predictions in terms of drug and target similarities and known DTIs. LI-DTI achieves comparable predictive performance to much more complex state-of-the-art baselines in all our experiments, including a prospective evaluation in which the models are trained using DTIs and similarity matrices obtained from the 2011 version of DrugBank (v3.0), and tested on DTIs obtained from the 2022 release of DrugBank (v5.0).

## 2 Methods

Several authors have shown that similarity measures between drugs and targets can be exploited for DTI prediction. For instance, drugs with similar chemical structures or side effects are likely to bind to similar protein targets. Our key challenge is to effectively utilize these similarities to generate accurate predictions while ensuring interpretability.

Recommender systems, particularly neighborhood-based collaborative filtering algorithms, are a class of algorithms specifically designed to make predictions by leveraging similarity relationships in data. Starting from a n×m matrix of ratings *R*, which contains a few known ratings of *n* users for *m* items, these algorithms predict missing ratings by leveraging two key principles: (i) users that are similar to each other have similar patterns of rating behavior, and (ii) items that are similar tend to receive similar ratings.

The drug-target prediction problem can be framed as a recommendation problem. We construct a n×m binary matrix *X*, where each row corresponds to one of the *n* drugs and each column corresponds to one of the *m* possible targets. If an interaction between drug *i* and target *j* is already known experimentally, we assign a value of 1 to the (i,j) entry in the matrix, and all other entries in the matrix are filled with zeros. The prediction problem then becomes identifying which zeros should be converted to ones. Notably, for a new drug (or a new target) an entire row (or column) of *X* may contain only zeros. This situation, known as “cold-start”, is particularly difficult and we will discuss it in detail later.

User-based nearest neighbor methods predict unknown ratings as a linear combination of the ratings given to the same item by similar users (Charu 2016). Formally, the predicted rating matrix $\hat{X}$ is obtained as:


(1) $$\hat{X}=(M_{U}\;{}^{\circ}\;S_{U})X$$

where $S_{U}$ is a zero-diagonal similarity matrix in which the (i,j) entry contains a similarity between users *i* and *j*, and $M_{U}$ is a masking matrix that retains only the top values in $S_{U}$ (i.e. the strongest user similarities) while setting all others to zero. Similarly, item-based nearest neighbor methods predict ratings as a linear combination of the ratings of the same user on similar items:


(2) $$\hat{X}=X(M_{I}\;{}^{\circ}\;S_{I})$$

where $S_{I}$ is a zero-diagonal matrix in which the (i,j) entry contains a similarity between items *i* and *j*, and $M_{I}$ is a masking matrix that retains only the top values in $S_{I}$ (i.e. the strongest item similarities) and sets all remaining entries to zero.

In standard user-based (or item-based) nearest neighbor methods, the similarity matrix $S_{U}$ (or $S_{I}$) is computed using predefined similarity measures over the rows (or columns) of *X*; for example, using cosine similarity, $S_{U}=X_{c}X_{c}^{T}$ where $X_{c}$ is the column-normalized matrix of *X* (so that each column has unit norm). Alternatively, in user-based (or item-based) nearest neighbor regression methods, the values of $S_{U}$ (or $S_{I}$) are learned from the data by minimizing an objective function *J*, with respect to $S_{U}$ (or $S_{I}$). A common choice for *J* is:


(3) $$J=||X-\hat{X}|{|}_{F}^{2}$$

where $||.|{|}_{F}^{2}$ indicates the Frobenius norm of a matrix. Often a regularization term is also added to the objective function *J*, to reduce overfitting.

The drug target prediction problem can be approached with user-based (or item-based) nearest neighbor methods, using our matrix *X* to calculate zero-diagonal matrices $S_{U}$ and $S_{I}$ of similarities between drugs and targets and then applying Equation (1) [or Equation (2)]. At the same time, we want to integrate additional information, not directly derivable from *X*, that can be expressed as similarity measures between drugs and targets.

**Figure 1:**
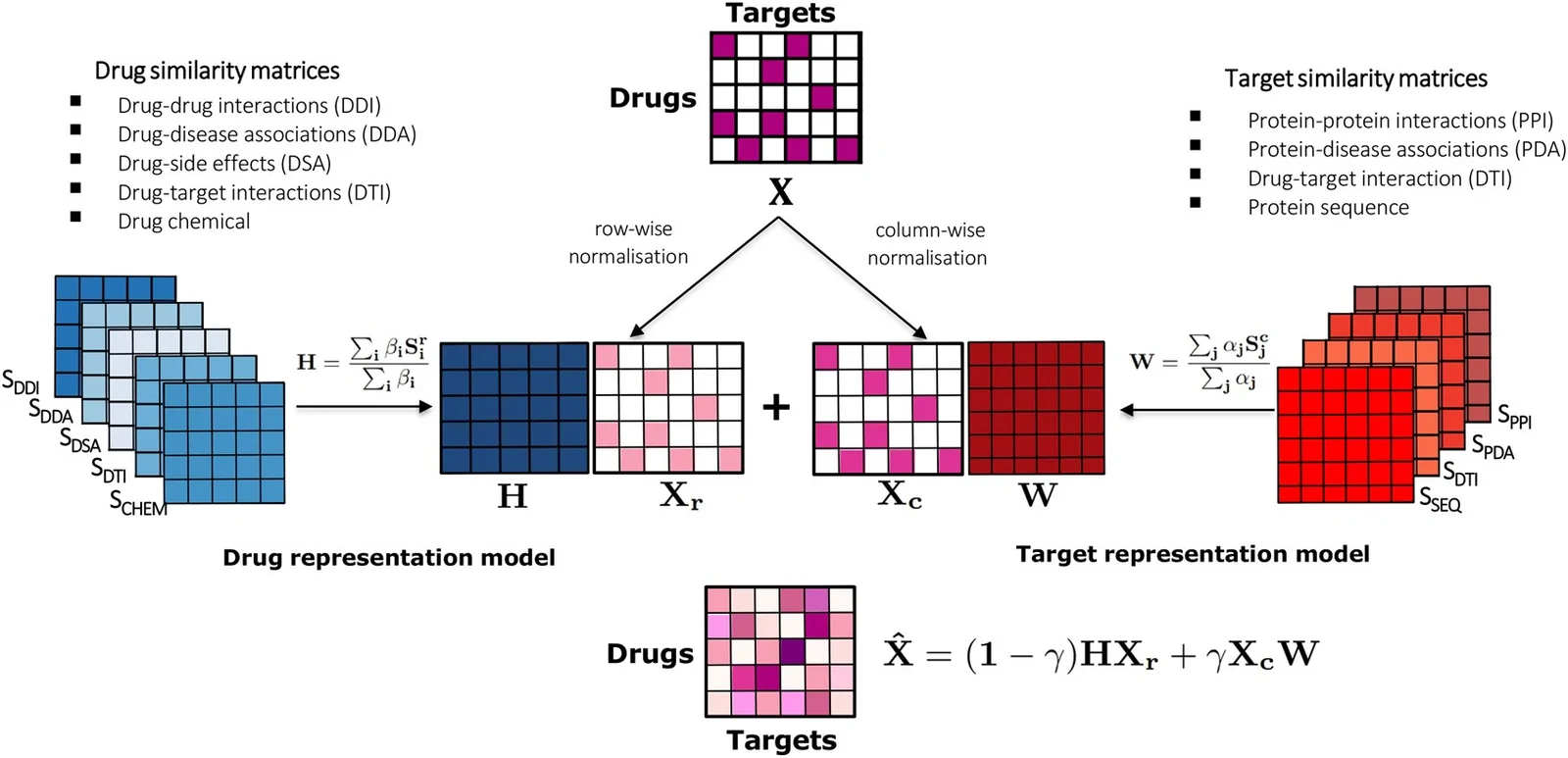
Overview of LI-DTI. DTIs are represented as an n×m matrix *X*, where observed interactions are encoded as 1 (coloured entries) and unobserved associations as 0. LI-DTI combines two linear models: the drug-based model, $HX_{r}$, represents each drug (row of X), as a linear combination of other drugs by learning a similarity matrix H that aggregates similarity matrices relating drugs (on the left); and the target-based model, $X_{c}W$, represents each target (column of X), as a linear combination of other targets by learning a similarity matrix W, that aggregates similarity matrices relating protein targets (on the right). Predictions are computed as $\hat{X}=(1-\gamma )HX_{r}+\gamma X_{c}W$, where the learned γ parameter controls the relative contribution of the two models.

LI-DTI is a linear combination of a user-based (drug-based) and an item-based (target-based) model, integrating similarity measures between drugs and between targets as a weighted sum:


(4) $$\hat{X}=(1-\gamma )HX_{r}+\gamma X_{c}W$$

where the parameter 0≤γ≤1 controls the relative contribution of each model. Matrices $X_{r}$ and $X_{c}$ are the row- and column-normalized versions of matrix *X* (see Fig. 1). Matrices *H* and *W* encode combined similarity information for drugs and targets and are built as weighted linear combinations of heterogeneous similarity matrices parameterized by non-negative coefficients ($\alpha_{c}$, $\alpha_{j}$, $\beta_{r}$, $\beta_{i}$). These coefficients are optimized during training and indicate how much each similarity source contributes to the final prediction:


(5) $$H=\frac{\beta_{r}X_{r}X_{r}^{T}+\sum_{i}\beta_{i}S_{i}^{r}}{\beta_{r}+\sum_{i}\beta_{i}}\;W=\frac{\alpha_{c}X_{c}^{T}X_{c}+\sum_{j}\alpha_{j}S_{j}^{c}}{\alpha_{c}+\sum_{j}\alpha_{j}}$$

where $S_{i}^{r}(i=1,..,Q)$ and $S_{j}^{c}(j=1,..,P)$ are zero-diagonal similarity matrices that encode side information about drugs (e.g. similarity in chemical structure or side effects) and targets (e.g. sequence similarity or target interactions). The term $X_{r}X_{r}^{T}$ (or $X_{c}X_{c}^{T}$) corresponds to a similarity matrix, like the one used in standard user-based (or item-based) neighbor methods [Equations (1) or (2)]. Thus, *H* is a linear combination of Q+1 similarity matrices, with *Q* matrices derived from heterogeneous information and one from the dataset *X* of known drug-target associations. A similar interpretation applies to matrix *W*. Similar to neighbor regression methods, the non-negative coefficients ($\alpha_{c}$, $\alpha_{j}$, $\beta_{r}$, $\beta_{i}$) are learned by minimizing an objective function:


(6) $$\begin{array}{c} \;\min_{\alpha ,\beta ,\gamma }\;J(\alpha ,\beta ,\gamma )={\left\|X-((1-\gamma )HX_{r}+\gamma X_{c}W)\right\|}_{F}^{2}\\ +\frac{\lambda_{1}}{2}\|\alpha_{c}{\|}_{F}^{2}+\frac{\lambda_{1}}{2}\|\beta_{r}{\|}_{F}^{2}+\frac{\lambda_{2}}{2}\|\alpha_{j}{\|}_{F}^{2}+\frac{\lambda_{2}}{2}\|\beta_{i}{\|}_{F}^{2} \end{array}$$

Therefore, LI-DTI is a linear combination of a drug-based and a target-based model, where each similarity matrix is itself a linear combination of multiple similarity matrices. Since all the parameters in the linear combination are constrained to be non-negative, the model offers a straightforward interpretation of the predictions in terms of how the different types of information contribute to the final prediction.

Importantly, LI-DTI can provide predictions even for cold-start cases, where a drug or a target has no previously known interactions, by relying on the guilt-by-association principle. For a drug *i* with no known targets, LI-DTI estimates the row *i* of the matrix *X* by aggregating the known interactions of the chemically similar drugs ($S_{i}^{r}$), weighted by their similarity to drug *i*. An analogous procedure is used for targets *j* using sequence similarity ($S_{j}^{c}$).

## 3 Materials

In our experiments, we used two DTI datasets. The first is the DTINet dataset, derived from the 2011 snapshot of DrugBank v3.0 as reported in Luo *et al.* (2017). This dataset contains 1923 interactions between 549 drugs and 424 human proteins (∼0.82% density). The second dataset is a prospective test set that we build from the 2022 snapshot of DrugBank (v5.0) in order to evaluate the performance in a more realistic scenario. This set comprises 514 new interactions between 181 drugs and 140 proteins that were not present in the original DTINet dataset. In all datasets, each drug and each protein has at least one known interaction. In Supplementary Information, we also report results for the DrugCentral (2022) and DrugBank datasets. In addition, we provide detailed information on all parameters used in our experiments.

### 3.1 Side information

For the drug and protein sets in the DTINet dataset, we used the side information compiled by Luo *et al.* (2017): (i) 3039 drug-drug interactions (DDI), (ii) 170 188 drug-disease associations (DDA), (iii) 65 240 drug-side effect associations (DSA), (iv) 600 protein-protein interactions (PPI), and (v) 476 457 protein-disease associations (PDA). Each type of side information is represented as a binary matrix, where rows correspond to drugs (or proteins), columns correspond to the associated entities (e.g. diseases or side effects), and an entry is equal to 1 if an association exists and 0 otherwise. All matrices share the same row indexing over the corresponding drug or protein set.

### 3.2 Drug similarities

For each of the DDI, DDA, and DSA matrices, we computed cosine similarity between all pairs of rows (drug profiles), resulting in the drug-drug similarity matrices $S_{\text{DDI}}^{d}$, $S_{\text{DDA}}^{d}$, and $S_{\text{DSA}}^{d}$, where the (i,j)-th entry contains the cosine similarity between drugs *i* and *j*. The matrix $S_{\text{DTI}}^{d}$ was computed using the same procedure applied to the rows of *X*. Drug chemical similarity $S_{\text{CHEM}}^{d}$ was computed using the Tanimoto coefficient on 2D molecular fingerprints.

### 3.3 Protein similarities

For PPI and PDA, we computed cosine similarity between all pairs of rows of the corresponding binary protein side information matrices, obtaining $S_{\text{PPI}}^{t}$ and $S_{\text{PDA}}^{t}$. The matrix $S_{\text{DTI}}^{t}$ was computed as the cosine similarity between columns of *X*. Note that, although both $S_{\text{DTI}}^{d}$ and $S_{\text{DTI}}^{t}$ are derived from the same DTI matrix *X*, they represent different similarities. Protein sequence similarity $S_{\text{SEQ}}^{t}$ was derived from normalized Smith-Waterman local alignment scores.

## 4 Results

**Figure 2:**
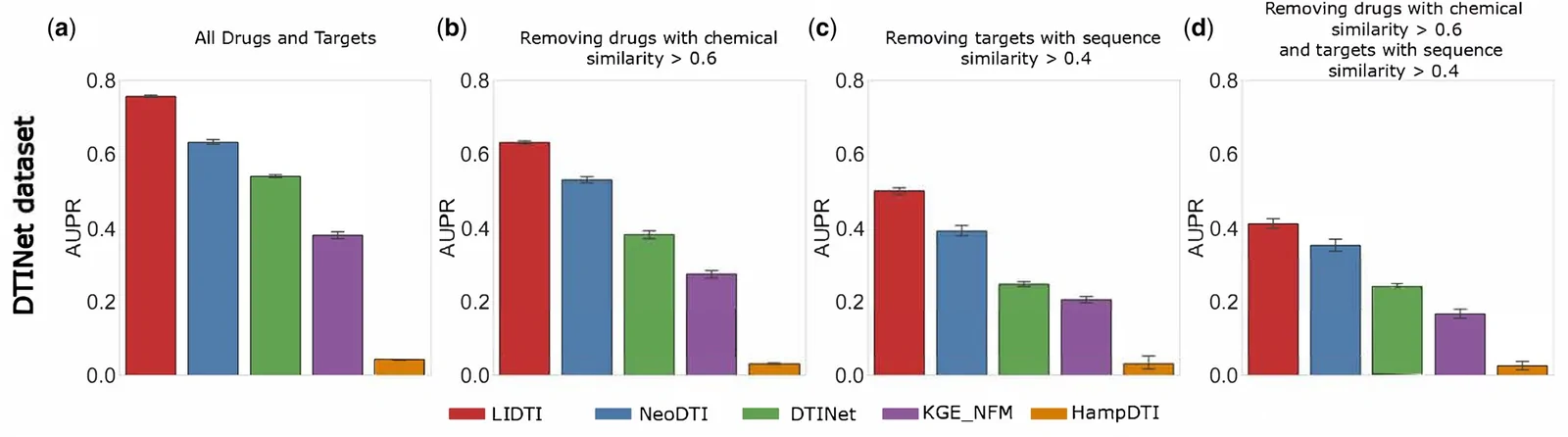
Performance comparison in warm start scenario. (a) Overall performance under 10-fold cross-validations 10 times. (b–d) Performance when controlling for similar drugs, targets and both drugs and targets, respectively.

We frame the DTI prediction problem as a binary classification task, where the goal is to predict the presence or absence of DTIs (Luo *et al.* 2017, Wan *et al.* 2019). Performance is primarily evaluated using the area under the precision–recall curve (AUPR), which is more informative than ROC analysis in highly imbalanced settings (Davis and Goadrich 2006). For completeness, AUROC results for competing methods are reported in Figs 1 and 2, available as supplementary data at *Bioinformatics* online.

As observed by Ye *et al.* (2021), practical DTI prediction settings often involve newly introduced drugs or targets with limited or no prior interaction annotations, requiring models to generalize beyond previously observed interactions. To reflect these practical use cases, predictive performance is evaluated under the following scenarios.

- *Warm Start*: In this scenario, all DTIs in the test set involve a drug and a target with at least one known interaction in the training set. This scenario answers the question *“Can we predict new interactions for drugs and targets that already have some known interactions?”*
- *Drug Cold-Start*: In this scenario, all DTIs in the test set involve a drug with no known interactions in the training set, and a target with at least one known interaction in the training set. This scenario answers the question *“Can we predict targets for a drug with no known interactions?”*
- *Target Cold-Start*: In this scenario, all DTIs in the test set involve a target with no known interactions in the training set, and a drug with at least one known interaction in the training set. This scenario answers the question *“Can we predict drugs targeting a protein without known interactions?”*

All evaluations presented in this section were performed on the DTINet dataset using 10-fold cross-validation. Constructing training and test splits that correctly reflect these scenarios is non-trivial; we therefore provide the sampling procedures and implementation details in the Supplementary Algorithms.

### 4.1 Warm start scenario

We compare the performance of LI-DTI with competing methods using a 10-fold cross-validation procedure, where 10% of the interacting and non-interacting DTIs are randomly selected as a test set. Importantly, the ratio of negative to positive samples in the test set is preserved, rather than undersampling negatives, a practice known to artificially inflate predictive performance (Krichene and Rendle 2020). During sampling, we ensure that every drug and target retains at least one known interaction in the training set. Model performance is reported as the mean AUPR across the 10 cross-validation runs. As shown in Fig. 2a, LI-DTI achieves an average AUPR of 0.7562 ± 0.0056 (mean and std.), obtaining the highest performance, and demonstrating improved recovery of unknown DTIs.

Because LI-DTI relies on similarities between drugs and between targets, an important question is how much its performance depends on having, in the training set, drugs that are similar to those in the test set, or targets that are homologous to test-set targets.

To assess the impact of chemical similarity, following Luo *et al.* (2017) we removed from the test set any DTI whose drug exhibited chemical similarity greater than 0.6 to a drug in the training set. Under this setting, LI-DTI achieved an average AUPR of 0.6309 ± 0.0074 (Fig. 2b), indicating effective recovery of missing interactions for chemically dissimilar drugs. To control for target homology, we excluded DTIs whose targets shared sequence similarity greater than 0.4 with any training target. In this scenario, LI-DTI achieved an average AUPR of 0.4991 ± 0.0015 (Fig. 2c) and remained effective at recovering interactions even for targets that are different in sequence from those observed during training. Finally, we controlled for both chemical and sequence similarity by excluding DTIs satisfying either criterion. This most challenging setting yielded an average AUPR of 0.4118 ± 0.0222 (Fig. 2d), indicating that LI-DTI retains predictive performance even when neither drugs nor targets are closely related to those observed during training.

### 4.2 Drug Cold-Start scenario

**Figure 3:**
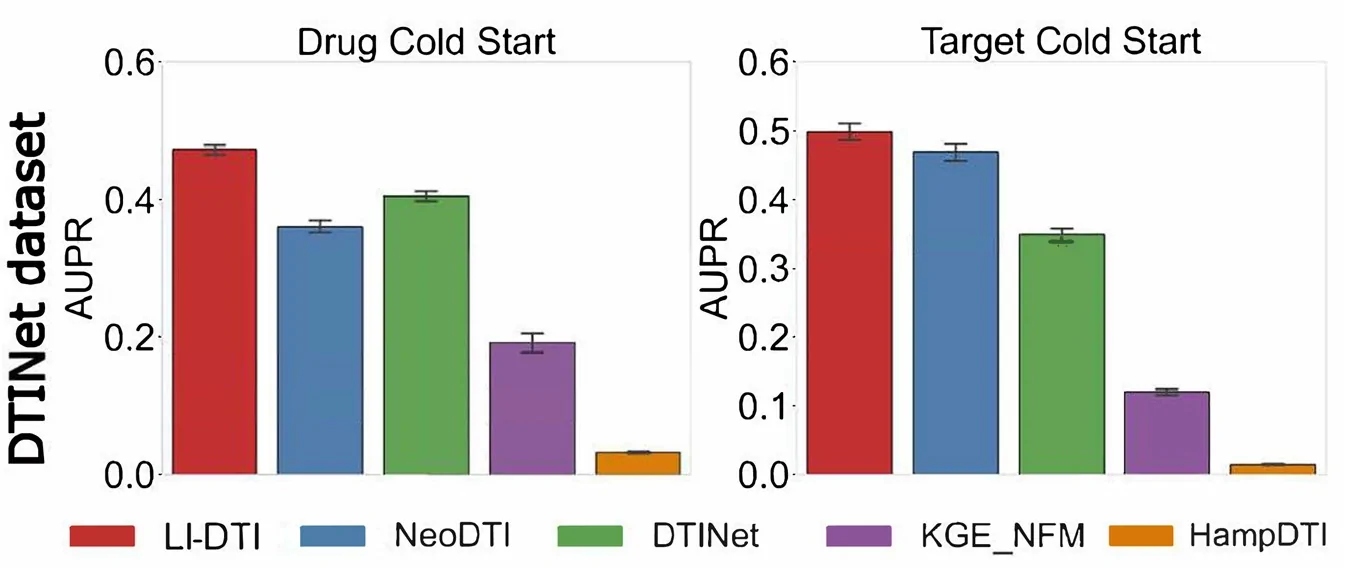
Performance in terms of AUPR in cold start scenarios. (a) Prediction of protein targets for drugs with no known interacting targets. (b) Prediction of drugs for proteins with no known drug interactions. In both cases, LI-DTI outperforms competitors.

In each cross-validation fold, all DTIs involving 10% of the drugs were assigned to the test set, while ensuring each remaining target retained at least one training interaction. LI-DTI achieved an average AUPR of 0.473 ± 0.0132, corresponding to the highest performance (Fig. 3a). Indicating that LI-DTI can effectively prioritize candidate targets for previously uncharacterized drugs, supporting its use in early-stage target identification for novel compounds.

### 4.3 Target Cold-Start scenario

In each cross-validation fold, all DTIs involving 10% of the targets were assigned to the test set, while ensuring each remaining drug retained at least one training interaction. LI-DTI achieved an average AUPR of 0.4988 ± 0.0206, again reaching the highest performance (Fig. 3b). These results indicate that LI-DTI remains effective at prioritizing candidate drugs for previously uncharacterized targets, supporting its potential for identifying therapeutic opportunities for newly studied or disease-associated proteins.

## 5 Prospective evaluation

We conducted a prospective evaluation by training our model on DTIs from the 2011 release of DrugBank (v3.0) and tested on novel DTIs introduced in the 2022 release of DrugBank (v5.0) that were absent from the DTINet dataset. This setup preserves the chronological order in which information becomes historically available and allows us to assess whether methods could have anticipated interactions confirmed years later. This prospective set contains 514 novel interactions involving 181 drugs and 140 proteins. Performance was measured using recall at top-*N* predictions (with N=10,20,50,100). For each drug (or target), we measured the fraction of its true novel interactions that are ranked among the top-*N* predicted candidates. This metric captures the proportion of true novel DTIs appearing among the most confidently predicted interactions, reflecting experimental prioritization utility.

**Figure 4:**
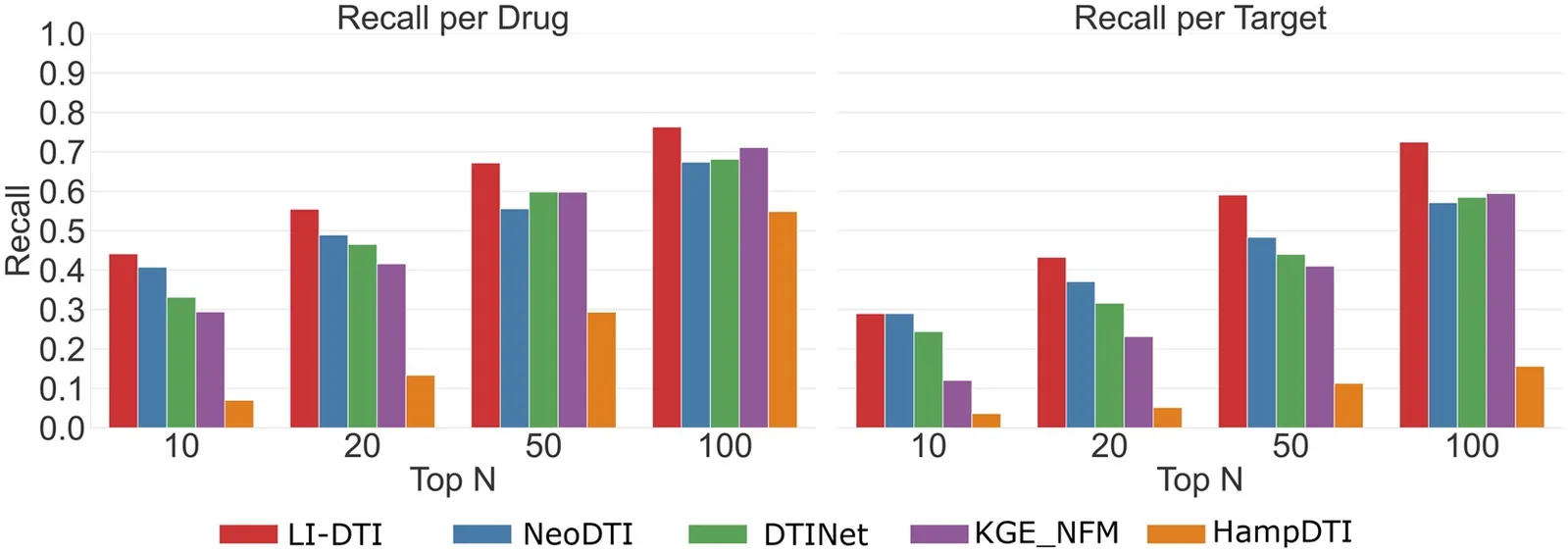
Prospective evaluation. We train each model with DTIs obtained from DrugBank in 2011 and test the performance on interactions added through 2022 in the top 10, 20, 50, and 100 predictions.

Figure 4 shows that LI-DTI consistently achieves higher recall than competing methods in both drug- and target-level evaluations. Improvements are most pronounced at larger *N* values, indicating that LI-DTI effectively ranks true interactions near the top of its predictions. Recall is generally lower in target-level evaluation, which is expected because often targets are associated with fewer known drugs than drugs are associated with known targets. These results suggest that many of the interactions later added to DrugBank could have been anticipated by LI-DTI already in 2011, highlighting its value for guiding experimental validation and drug discovery. To complement our analysis, we globally assessed the side-information contributions across the top 20 000 predictions in the DTINet dataset. Figure 4, available as supplementary data at *Bioinformatics* online shows the number of times each drug and target side information are used in the predictions as well as the total contribution of each source. Similarities derived from known interactions contribute most strongly to the predictions, although similarities derived from biological evidence, particularly PPI networks and sequence similarity, also provide substantial support for the predictions.

## 6 Interpretability

The core contribution of LI-DTI is the ability to provide inherently interpretable predictions while maintaining high predictive performance. Because LI-DTI is a linear model, each predicted interaction score can be decomposed into contributions from different similarity sources. This allows us to trace a DTI prediction back to specific contributions at different levels.

**Figure 5:**
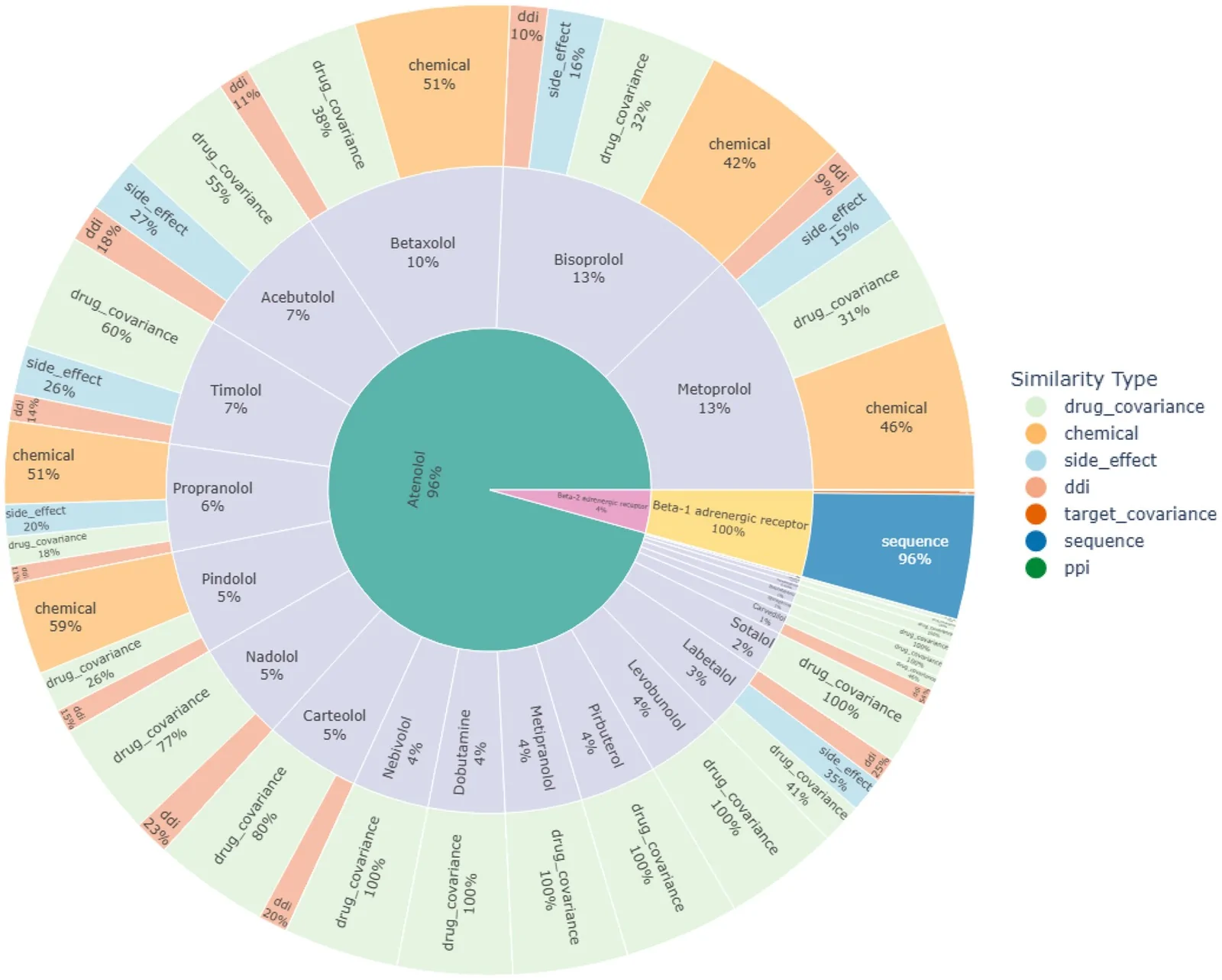
Explanation of Atenolol-ADRB2 interaction predicted scores. The final score for a DTI prediction can first be interpreted in terms of the drug and target representation models. These contributions are represented by the innermost levels in the sunburst plot. The next level shows the aggregated similarities of each drug for the drug representation model and of each target for the target representation model. The last and outermost level shows the breakdown of each type of side information used to calculate the similarities.

To illustrate this capability, we consider one of the top interaction predictions produced by LI-DTI: the beta-blocker Atenolol and the target ADRB2 in the prospective set (2022). The sunburst plot in Fig. 5 provides a detailed breakdown of how LI-DTI arrives at this prediction. As defined in Equation (4), LI-DTI computes prediction scores as a linear combination of a drug-based and a target-based model. The central (root) node of the sunburst plot shows that 96% of the predicted score is explained by the drug-based model (green), while the remaining 4% is explained by the target-based model (pink). The second ring shows the contributions of the different types of side information. On the drug side (teal), we can see the contribution of the similarities between Atenolol and other drugs known to target ADRB2 to the drug-based score. Notably, other beta-blockers, namely Metoprolol, Bisoprolol, and Betaxolol, account for about 36% of the score, primarily due to their high chemical similarity to Atenolol (shown in orange in the outermost ring). On the target side (yellow), the contribution arises from similarities between ADRB2 and Atenolol’s only known target in the 2011 dataset, ADRB1. Consistent with the global contribution analysis, the outermost ring indicates that the contribution is driven mainly by sequence similarity (96%). Another example is shown in Fig. 3, available as supplementary data at *Bioinformatics* online, which details the predicted Levonorgestrel-glucocorticoid interaction.

To facilitate exploration of the predictions, we provide a web tool where experimentalists can search for drugs and targets and generate sunburst plots for LI-DTI predicted scores at https://paccanarolab.org/lidtiweb/. The tool is available for the latest release of DrugBank (2025).

## 7 Discussion

Recent knowledge graphs and deep learning approaches for DTI prediction can achieve strong predictive performance, but often operate as black boxes with limited interpretability. The fundamental question addressed in this work is whether a substantially simpler linear model can achieve competitive predictive performance. Such a model would have the clear advantage of being inherently interpretable, enabling direct inspection of how predictions are generated.

We present LI-DTI, a linear and interpretable machine learning model for predicting unknown DTIs. Like existing approaches, LI-DTI leverages heterogeneous similarity information about drugs and targets; however, its linear formulation preserves transparency and allows predictions to be directly related to the underlying sources of biological information. Extensive evaluation on standard benchmark datasets, as well as on a novel prospective dataset introduced in this work, shows that LI-DTI achieves strong predictive performance without sacrificing interpretability. Importantly, LI-DTI remains applicable in cold-start settings where it achieves state-of-the-art performance. The performance decrease relative to the warm-start setting, reflects the greater difficulty of predicting interactions for unseen drugs and targets in the absence of direct interaction profile information. Thanks to its linear formulation, LI-DTI prediction scores can be decomposed into contributions from different similarity sources on both the drug and target sides. For experimentalists, this decomposition is particularly valuable, as it reveals which sources of biological evidence support a given prediction.

To facilitate exploration of predicted DTIs, we provide a web tool where researchers can inspect how similarity sources contribute to each prediction. Importantly, candidate interactions to which LI-DTI does not assign top scores may still represent strong biological hypotheses when their predicted scores are supported by coherent and interpretable evidence. This interpretability distinguishes LI-DTI from existing approaches and enables experimentalists to prioritize candidates not only based on rank, but also on the underlying biological rationale, leading to more informed decisions.

More broadly, we believe that the machine learning principles underlying LI-DTI can be applied to other biological problems, and potentially to other domains. In particular, LI-DTI naturally extends to problems involving the prediction of associations between pairs of entities, as encountered in problems like drug-side effect prediction and disease-gene association prediction, where heterogeneous similarity information is available and interpretability is a key requirement.

## Supplementary Material

btag428_Supplementary_Data

## Data Availability

The data underlying this article are available in the Zenodo repository, at https://zenodo.org/records/18262393.
