# Supplementary material for: A linear interpretable model for drug target prediction
Source: Bioinformatics. 2026 Aug 21;42(Suppl 2):btag428. doi: 10.1093/bioinformatics/btag428 (PMC13501308; doi:10.1093/bioinformatics/btag428)
Supplement: Supplementary file 1 [file btag428_supplementary_data.pdf]

Supplementary Information:

A linear interpretable model for Drug Target Prediction

Santiago Noto,[1,2] Santiago Ferreyra,[1] Ruben Jimenez,[1,2] Diego Galeano,[1,2] and Alberto Paccanaro[1,2,*].

1. School of Applied Mathematics, Fundação Getulio Vargas, Rio de Janeiro, Brazil
2. Department of Computer Science, Centre for Systems and Synthetic Biology, Royal Holloway, University of London, Egham Hill, Egham, UK

∗ Corresponding author. Alberto Paccanaro, alberto.paccanaro@rhul.ac.uk

Contents

1. Supplementary Figures
    a. Supplementary Figure S1 2
    b. Supplementary Figure S2 2
    c. Supplementary Figure S3 3
    d. Supplementary Figure S4 4
2. Distinction from Affinity-Based DTI Models 5
3. Hyperparameters used in our experiments 6
4. Supplementary Algorithms
    a. Warm-Start Sampling Procedure 7
    b. Cold-Start Sampling Procedure 8
5. Supplementary Results
    a. Warm-Start Scenario 9
    b. Cold-Start Scenario 10
    c. Prospective Evaluation 11
    d. Additional Evaluation 12
6. References 13

1. Supplementary Figures

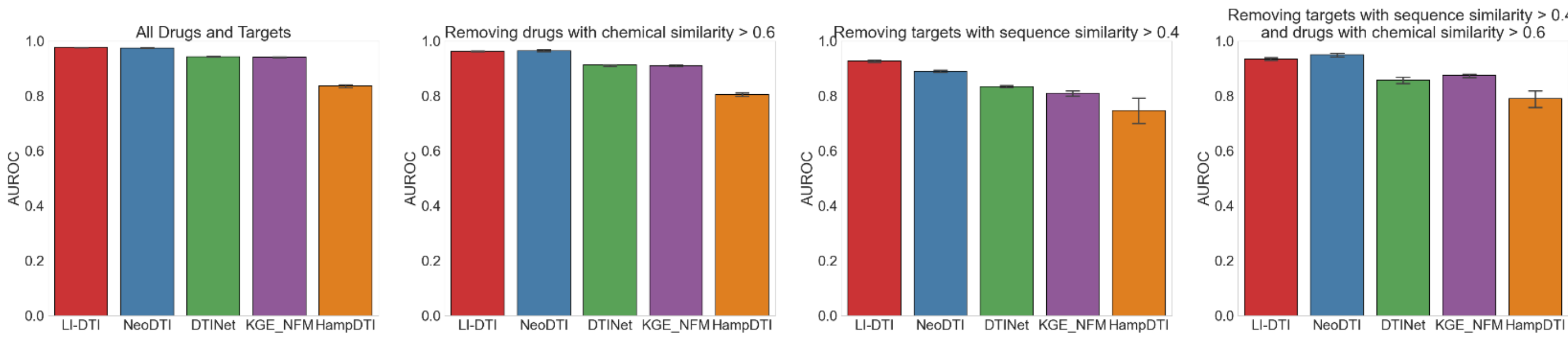

Supplementary Figure S1. *Performance comparison of AUROC in the warm start scenario for varying degrees of drug and target similarity on the DTINet dataset. a) Overall performance on the entire dataset when running 10-fold cross-validations 10 times using the DTINet dataset. b, c, d) Performance in the DTINet dataset when controlling for similar drugs, targets and both drugs and targets, respectively. The corresponding AUROC values are reported in the Supplementary Tables.*

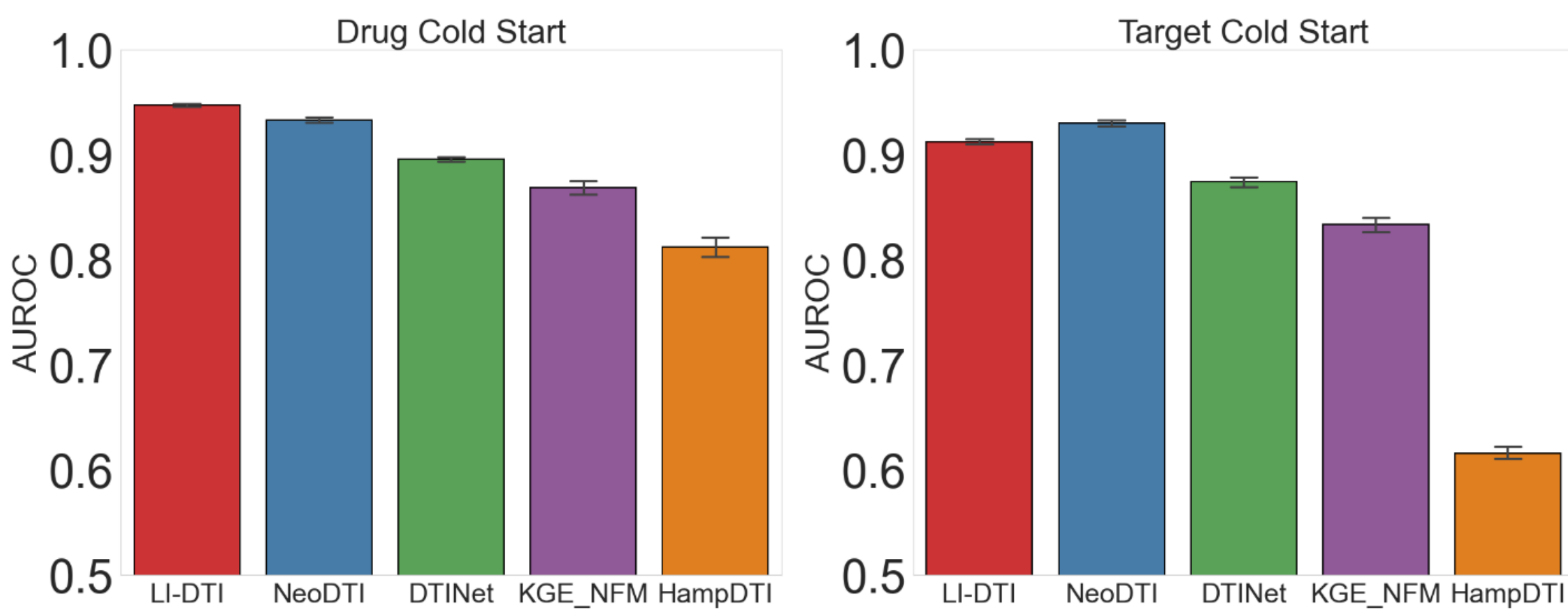

Supplementary Figure S2. *Performance in terms of AUC in cold start scenarios for drugs and targets. a) LI-DTI shows state-of-the-art performance when trying to predict potential protein targets for drugs with no previous known interacting targets. b) Given a protein with no known drug interactions, can predict drugs that will target that protein. The corresponding AUROC values are reported in the Supplementary Tables.*

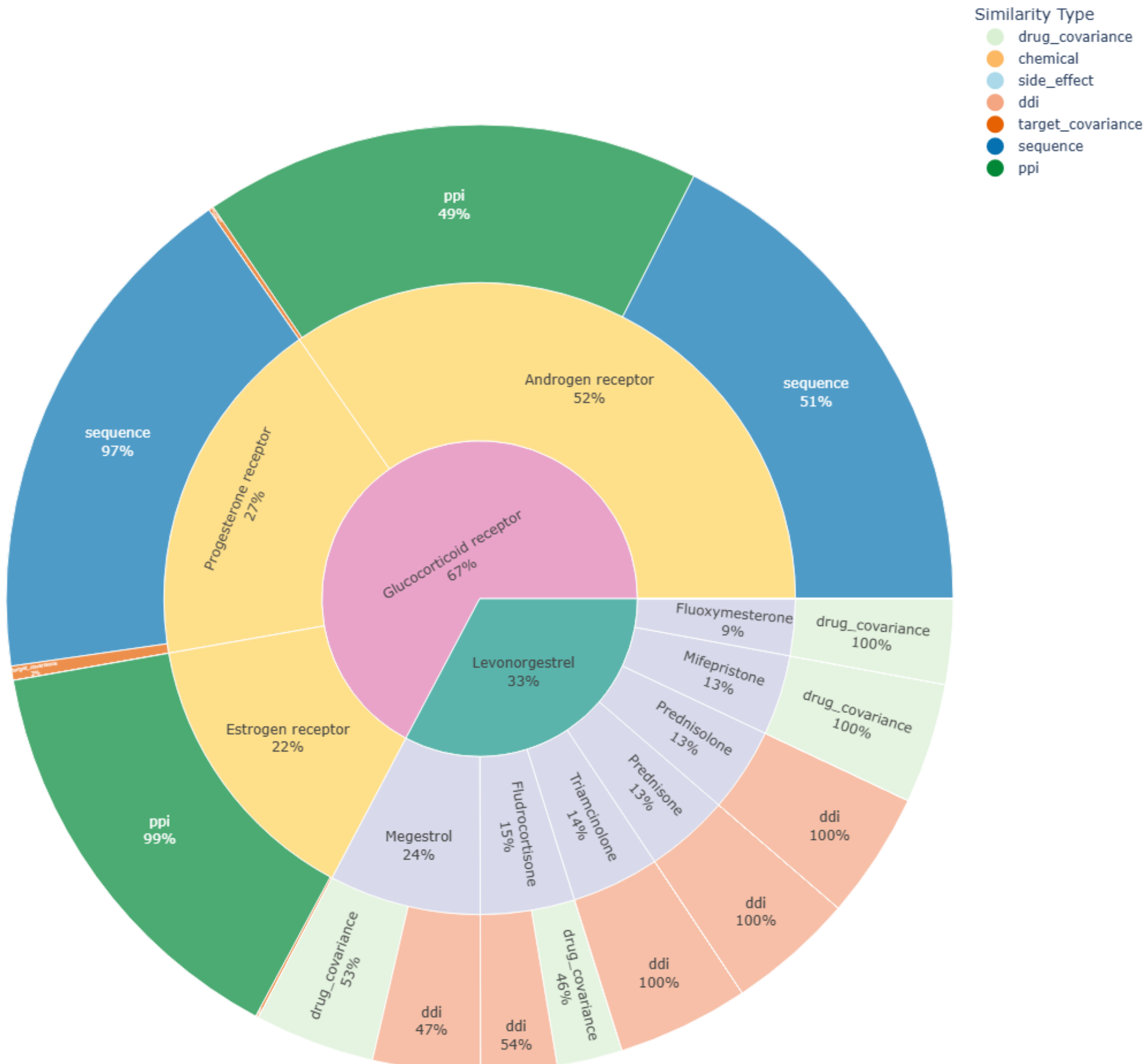

Supplementary Figure S3. *Explanation of the Levonorgestrel-Glucocorticoid receptor DTI. Sunburst plot provides the breakdown of the score for this DTI. Colors in the outer ring indicate similarity type, whereas inner-ring colors are used to distinguish the hierarchical decomposition of the target- and drug-side contributions. The drug representation model accounts for 33% of the total score and the target representation model accounts for 67%. This is the 15th ranked DTI in the prospective set. On the target side (center in pink), the prediction is mainly explained by contributions from the Estrogen receptor, Progesterone receptor, and Androgen receptor (yellow sectors), whose influence arises primarily from sequence similarity and protein–protein interaction (PPI) evidence (outer blue and green sectors). On the drug side (center in green), the prediction is explained by contributions from Megestrol, Fludrocortisone, Triamcinolone, Prednisone, Prednisolone, Mifepristone, and Fluoxymesterone (gray sectors), derived mainly from drug covariances and drug–drug interactions (outer light-green and salmon sectors).*

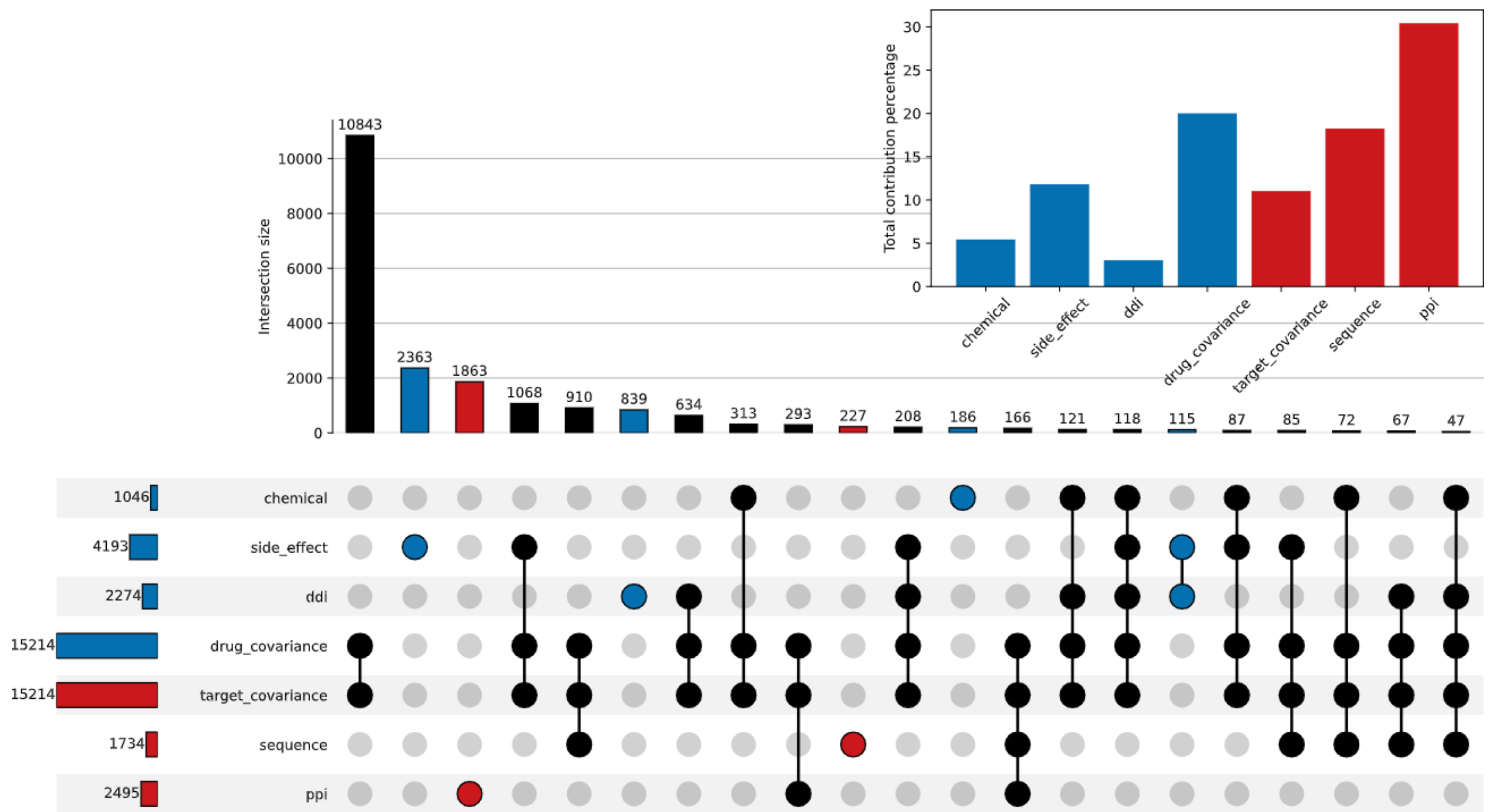

Supplementary Figure S4. *Global contribution of the similarities to the top 20,000 predictions in the DTINet dataset. The horizontal bars show the number of times each drug side information (blue) and target side information (red) are used in the top 20,000 predictions. The vertical bars, together with the painted circles, show how many times a given combination of the side effects appear together in these predictions. The barplot at the top right shows the sum of the contributions per side effect across the top 20,000 predictions. The figure reports (i) how frequently each type of drug-side and target-side information contributes to predictions and (ii) their relative contribution to the overall prediction scores. DTI-based similarities (covariances) on both the drug and target sides appear most frequently, indicating that many predictions are supported by comparisons to known interaction profiles. In contrast, target-side biological information, particularly protein-protein interaction (PPI) similarity and protein sequence similarity. We restricted the combinations to appear on at least 40 predictions.*

2. Distinction from Affinity-Based DTI Models

Recently, deep learning architectures have also been proposed for drug-target interaction prediction, including MolTrans [1] and DrugBAN [2]. These methods address a related but not identical prediction task. In these approaches, both training and evaluation are typically based on large-scale binding-affinity datasets derived from resources such as BindingDB and ChEMBL [3], where binary interaction labels are constructed by thresholding experimental affinity measurements. Thus, these models use a different operational definition of drug-target interaction from the one considered in our work, that focuses on DrugBank which provides drug–target annotations supported by expert evaluation and literature evidence.

Converting binding-affinity measurements into binary interaction labels is not straightforward, because there is no unique or universally accepted way to threshold and combine these data. BindingDB and ChEMBL compile binding affinity measurements obtained using several experimental quantities, most commonly Ki, Kd, IC50 and EC50 [4]. In many computational studies, a drug–target pair is considered to interact when at least one of these measurements is below a predefined threshold, such as 10 μM. However, these quantities describe different biological and biochemical properties. The dissociation constant Kd measures the affinity between a ligand and its target. Ki and IC50 are inhibition-related quantities associated with the concentration required to reduce enzymatic activity under specific experimental conditions. EC50, in contrast, represents the concentration required to achieve 50% of the maximum biological response in a functional assay. Because these measurements are derived from different experimental settings and capture distinct biological properties, directly combining them into a single interaction definition can introduce noise.

Kalliokoski et al. [5] point out that IC50 values obtained across different experiments are often not directly comparable, and that publicly available data may contain inconsistencies and errors. Although a threshold such as 10 μM is commonly adopted in computational studies, this cutoff is not universally established. MolTrans and DrugBAN also reflect this issue by defining positive interactions as those with IC50 < 100 nM and negative interactions as those with IC50 > 10,000 nM, thereby excluding an intermediate range of measurements.

The difference in problem formulation affects the scale of the resulting datasets. For example, the reduced version of BindingDB used by DrugBAN contains 14,643 drugs, 2,623 proteins and 49,199 interactions. These numbers are substantially larger than those available in the DrugBank-derived datasets. The smaller number of curated DrugBank [6] interactions makes these datasets less suitable for training large deep learning architectures from scratch but also reflects the different objective of our work: predicting curated drug-target interaction annotations rather than thresholded binding-affinity labels. For these reasons, we consider methods such as MolTrans and DrugBAN to be important recent methods for a related, affinity-derived prediction setting, but not directly comparable baselines for the DrugBank-derived curated interaction setting considered in this work.

3. Hyperparameters used in our experiments

LI-DTI learns a small set of non-negative coefficients, rather than high-dimensional model parameters, controlling the relative contribution of each drug-side and target-side similarity source, together with the parameter γ, which balances the drug-based and target-based components of the prediction. These coefficients are optimized using MATLAB's *fmincon* with the *trust-region-reflective* algorithm. Since the objective is solved with a local constrained optimizer, convergence should be interpreted as convergence to a local stationary solution rather than as a formal guarantee of global optimality. In our experiments, we did not observe instability across cross-validation runs.

- **Learnable weights**. The optimal values of alpha_c, alpha_j, beta_r, beta_i,gamma in were obtained by minimizing the objective function using MATLAB's fmincon optimizer with the trust-region reflective algorithm.
- **Hyperparameters**. The regularization penalties for our experimental procedures were set to lambda_1 = 1.0 and lambda_2 = 0.1.

<u>Procedure of similarities</u>

- The drug chemical sequence similarities we used the SMILES representations to obtain a daylight-like fingerprint with RDKit using the FingerprintMol function with the default parameters (minPath=1, maxPath=7, fpSize=2048, bitsPerHash=2, minSize=128).
- The protein sequence similarities were obtained using the normalized Smith-Waterman score calculated using MATLAB function swalign, with the scoring matrix BLOSUM62.
- All similarity values in each of the similarity matrices, S_i^r and S_j^c, are bounded between 0 and 1. We set thresholds for these matrices, below which the values of the matrices were set to zero. These thresholds were set to:
  (i) drug-drug interaction (DDI) similarities (0.7),
  (ii) drug-disease (DDA) similarities (0.0),
  (iii) drug-side effect (DSA) similarities (0.5),
  (iv) protein-protein (PPI) similarities (0.4),
  (v) protein-disease (PDA) similarities (0.0),
  (vi) drug chemical similarities (0.6), and
  (vii) target sequence similarities (0.3).
- **Competitors**. Each method was retrained on the DTINet dataset and evaluated using the same 10-fold cross-validation protocol. We used the official code released by the authors, with hyperparameters set according to the guidelines provided in their respective publication.
  (1) **DTINet**: embedding size 150, restart probability 0.5, rank 40 and regularization 10;
  (2) **NeoDTI**: embedding size 1024;
  (3) **KGE_NFM**: learning rate 0.001, pairwise loss, regularization loss 1e-5, regularizer norm 3, layer number and units in each layer 128, l2 reg. 1e-5 (warm-start), and 1e-3 (cold-start), batch size 10,000; and
  (4) **HampDTI**: embedding size 128, meta-path length 3, number of channels 4, balance coefficient 0.4.

4. Supplementary Algorithms

Warm Start Sampling Procedure

The algorithm takes as an input the DTI matrix **X ∈ ℝ^(drugs × targets)**, and outputs a matrix **Test** ∈ **ℝ^(drugs × targets)**, such that $\mathbf{Test_{ij} = 1}$ if the DTI between drug *i* and target *j* can be removed from **X** without causing a cold start for the drugs or the targets.

The first part (lines 1–3) retrieves all the known interactions in **X**. The function **randperm(n)** gives a random permutation of the numbers from 1 to *n*, and the function **sum(X)** gives the sum of all the elements in **X**, which is the number of known DTIs. This is used to iterate through the known DTIs in a random order to check which DTIs can be removed from the matrix **X** without causing cold start for the drugs or the targets.

To check for cold start cases, it temporarily removes each DTI from the matrix **X** and checks if the number of elements in the column (**sum(sumCols(tmp) == 0) > 0**) and row (**sum(sumRows(tmp) == 0) > 0**) of the removed DTI is 0. If either of them is 0, removing the DTI causes cold start, so it cannot be removed. If neither is 0, then it removes the DTI from the DTI matrix **train** and adds it to the **Test** matrix.

Once the iterations are done, the matrix **Test** contains a set of DTIs that can be used for testing the warm-start scenario. In the final step, the matrix **train** contains DTIs that, if removed, will cause a cold start for either the drugs or the targets. Neither of these DTIs can be used for testing and are always used for training.

Algorithm Steps

- **Input: DTI matrix X ∈ ℝ^(drugs × targets)**
- **Output: Test ∈ ℝ^(drugs × targets)**

```
1.  train ← X
2.  Test ← zeros(size(X))
3.  [Rows, Cols] ← find(X)
4.  for i in randperm(sum(X)) do
5.      tmp ← train
6.      tmp[Rows[i], Cols[i]] ← 0
7.      if sum(sumCols(tmp) == 0) > 0 or sum(sumRows(tmp) == 0) > 0 then
8.          Continue
9.      else
10.         train[Rows[i], Cols[i]] ← 0
11.         Test[Rows[i], Cols[i]] ← 1
12.     end if
13. end for
```

Cold Start Sampling Procedure

Proposed Algorithm to obtain valid drugs for drug cold start or target cold start evaluation. The algorithm takes as an input the DTI matrix $\mathbf{X} \in \mathbb{R}^{(\text{drugs} \times \text{targets})}$, and outputs a vector **Test** $\in \mathbb{R}^{(\text{drugs})}$, such that $\mathbf{Test}_i = 1$ if the drug (row) *i* can be removed from **X** without leaving a target with no known interaction.

It starts by sampling drugs (rather than DTIs). In the DTI matrix **X**, this is equivalent to sampling rows $\mathbf{X}_{i}*$ rather than elements $\mathbf{X}_{ij}$. The algorithm randomly iterates through the drugs (rows of the matrix, **randperm(number of drugs)**) and checks if the interactions of that drug can be removed without leaving a target with no known interactions.

This is done by setting the row *i* to 0 (**tmp[i, :] ← 0**) and checking if the number of elements in any column is 0 (**sum(sumCols(tmp) == 0) > 0**). If it is 0, removing the drug causes a cold start for at least one target, so it cannot be removed. If it is not 0, then it removes the DTIs from the drug matrix **train**, by setting all the values of the row to 0. Drug *i* is added to the **Test** vector (**Test[i] ← 1**).

Once the algorithm iterates over all the drugs, the vector **Test** contains a set of drugs that can be used for testing the drug cold-start scenario. In the final step, the matrix **train** contains DTIs that, if removed, will cause a cold start for the targets. Neither of these DTIs can be used for testing and are always used for training.

Algorithm Steps

- **Input: DTI matrix** $\mathbf{X} \in \mathbb{R}^{(\text{drugs} \times \text{targets})}$
- **Output: Test** $\in \mathbb{R}^{(\text{drugs} \times \text{targets})}$

1. **train ← X**
2. **Test ← zeros(number of drugs)**
3. **for** *i* in **randperm(number of drugs) do**
4.   **tmp ← train**
5.   **tmp[i, :] ← 0**
6.   **If** sum(sumRows(tmp) == 0) > 0
7.   Continue
8.   **else**
9.   **train[i, :] ← 0**
10.   **Test[i] ← 1**
11.   **end if**
12. **end for**

---

5. Supplementary Results

For the warm-start and cold-start experiments, we used the DTINet drug-target interaction dataset derived from the 2011 snapshot of DrugBank v3.0. This dataset was used to evaluate LI-DTI. We retained only drugs and targets with at least one known interaction, resulting in a curated interaction matrix containing 1,923 drug-target interactions between 549 drugs and 424 human proteins.

1. Warm-Start Scenario

Method	AUROC		AUPR	
	**mean**	**std**	**mean**	**std**
**LI-DTI**	**0,97737**	0,00117	**0,75617**	0,00559
**NeoDTI**	0,97518	0,00158	0,63139	0,01062
**DTINet**	0,94304	0,00190	0,53876	0,00684
**KGE_NFM**	0,94165	0,00231	0,38088	0,01672
**HampDTI**	0,83709	0,00967	0,04399	0,00383

- *Removing chemical similarity > 0.6.*

Method	AUROC		AUPR	
	**mean**	**std**	**mean**	**std**
**LI-DTI**	0,9629	0,00267	**0,6310**	0,00741
**NeoDTI**	**0,9653**	0,00530	0,5288	0,01468
**DTINet**	0,9127	0,00520	0,3826	0,01805
**KGE_NFM**	0,9107	0,00471	0,2753	0,01685
**HampDTI**	0,8060	0,01167	0,0312	0,00364

- *Removing sequence similarity > 0.4.*

Method	AUROC		AUPR	
	**mean**	**std**	**mean**	**std**
**LI-DTI**	**0,9502**	0,00383	**0,4992**	0,01443
**NeoDTI**	0,9495	0,00426	0,3922	0,02169
**DTINet**	0,8815	0,00618	0,2476	0,01085
**KGE_NFM**	0,9006	0,00451	0,2057	0,01379
**HampDTI**	0,7993	0,04140	0,0313	0,03108

- *Removing chemical similarity > 0.6 and sequence similarity > 0.4.*

Method	AUROC		AUPR	
	mean	std	mean	std
LI-DTI	0,9348	0,00881	**0,4119**	0,02110
NeoDTI	**0,9499**	0,01096	0,3532	0,02708
DTINet	0,8581	0,01748	0,2421	0,01168
KGE_NFM	0,8744	0,00998	0,1670	0,02042
HampDTI	0,7904	0,05210	0,0258	0,01882

2. Drug Cold-Start Scenario

Method	AUROC		AUPR	
	mean	Std	mean	std
LI-DTI	**0,9474**	0,0030	**0,4730**	0,0132
NeoDTI	0,9333	0,0040	0,3611	0,0149
DTINet	0,8962	0,0037	0,4057	0,0125
KGE_NFM	0,8686	0,0104	0,1920	0,0226
HampDTI	0,8120	0,0153	0,0323	0,0025

3. Target Cold-Start Scenario

Method	AUROC		AUPR	
	mean	std	mean	std
LI-DTI	0,9125	0,0042	**0,4988**	0,0206
NeoDTI	**0,9303**	0,0049	0,4695	0,0222
DTINet	0,8743	0,0081	0,3499	0,0163
KGE_NFM	0,8335	0,012	0,1195	0,0079
HampDTI	0,616	0,0102	0,0146	0,0016

4. Prospective Evaluation

For the prospective evaluation, we constructed an independent test set from a 2022 snapshot of DrugBank v5.0. This dataset contains 514 newly reported drug–target interactions involving 181 drugs and 140 proteins. Importantly, these interactions were not present in the DTINet dataset derived from the 2011 DrugBank v3.0 snapshot, allowing us to evaluate the ability of LI-DTI to recover drug–target associations that became available only after the original training data were compiled.

- *(Recall per Drug)*

Top-N	LI-DTI	NeoDTI	DTINet	KGE_NFM	HampDTI
**1**	0,1524	**0,1698**	0,0715	0,0458	0,0083
**3**	**0,2887**	0,2792	0,1703	0,1043	0,0083
**5**	**0,3560**	0,3412	0,2335	0,1772	0,0209
**10**	**0,4414**	0,4074	0,3312	0,2940	0,0699
**20**	**0,5545**	0,4892	0,4652	0,4159	0,1333
**50**	**0,6718**	0,5556	0,5984	0,5978	0,2934
**100**	**0,7630**	0,6739	0,6810	0,7109	0,5487

- *(Recall per Target)*

Top-N	LI-DTI	NeoDTI	DTINet	KGE_NFM	HampDTI
**1**	0,0534	**0,0839**	0,0402	0,0263	0,0000
**5**	0,2094	**0,2199**	0,1807	0,0576	0,0113
**10**	**0,2899**	**0,2899**	0,2441	0,1204	0,0361
**20**	**0,4327**	0,3707	0,3161	0,2314	0,0516
**50**	**0,5905**	0,4832	0,4398	0,4100	0,1129
**100**	**0,7247**	0,5711	0,5846	0,5944	0,1561

5. Additional Evaluations

Additionally, to further assess the generalizability of LI-DTI beyond the original DTINet benchmark, we report supplementary experiments on two larger and more recent curated drug-target interaction datasets. For both datasets, we used the same 10-fold cross-validation setting and the same sampling procedures as in the main experiments.

First, we evaluated LI-DTI in both warm-start and cold-start settings using the DrugCentral (2022) dataset [7]. This dataset contained 14,689 drug-target interactions involving 2,278 drugs and 1,646 human proteins, corresponding to a density of 0.39%. This evaluation allowed us to assess whether LI-DTI remains effective when predicting interactions involving previously unseen drugs or targets in a larger and independently curated dataset. In the warm-start setting, LI-DTI achieved an AUPR of **0.5920**. When individual similarity sources were removed, the AUPR was **0.5985** after removing chemical similarity, **0.5575** after removing sequence similarity, and **0.5698** after removing both chemical and sequence similarity. In the cold-start settings, LI-DTI achieved an AUPR of **0.1959** for drug cold-start and **0.4128** for target cold-start.

Second, we evaluated LI-DTI in the warm-start setting using a more recent DrugBank (2025) snapshot [6]. This dataset contained 15,956 drug-target interactions involving 6,451 drugs and 2,597 targets. This additional benchmark was included to evaluate the behavior of LI-DTI on a more recent version of DrugBank and to provide a broader comparison across datasets with different sizes, densities, and curation sources. On this dataset, LI-DTI achieved an AUC of **0.9451** and an AUPR of **0.4035**.

Together, these additional experiments support the robustness of LI-DTI and provide a more comprehensive assessment of its generalization ability beyond the original DTINet/DrugBank v3.0 benchmark.

6. References

[1] Huang, K., Xiao, C., Glass, L. M., & Sun, J. (2021). MolTrans: molecular interaction transformer for drug–target interaction prediction. *Bioinformatics*, *37*(6), 830-836.

[2] Bai, P., Miljković, F., John, B., & Lu, H. (2023). Interpretable bilinear attention network with domain adaptation improves drug–target prediction. *Nature Machine Intelligence*, *5*(2), 126-136.

[3] Wassermann, A. M., & Bajorath, J. (2011). BindingDB and ChEMBL: online compound databases for drug discovery. *Expert opinion on drug discovery*, *6*(7), 683-687.

[4] Chatterjee, A., Walters, R., Shafi, Z., Ahmed, O. S., Sebek, M., Gysi, D., ... & Menichetti, G. (2023). Improving the generalizability of protein-ligand binding predictions with AI-Bind. *Nature communications*, *14*(1), 1989.

[5] Comparability of Mixed IC50 Data – A Statistical Analysis — Kalliokoski T, Kramer C, Vulpetti A, Gedeck P (2013). Comparability of Mixed IC50 Data – A Statistical Analysis. PLOS ONE 8(4): e61007.

[6] DrugBank 6.0: the DrugBank Knowledgebase for 2024 — Knox C. et al. (2024). DrugBank 6.0: the DrugBank Knowledgebase for 2024. Nucleic Acids Research, 52(D1), D1265–D1275.

[7] Ursu, O., Holmes, J., Bologa, C. G., Yang, J. J., Mathias, S. L., Stathias, V., ... & Oprea, T. (2019). DrugCentral 2018: an update. *Nucleic acids research*, *47*(D1), D963-D970.
